# A global perspective on smoking’s impact on peptic ulcer disease: DALY trends and projections

**DOI:** 10.3389/fpubh.2025.1550045

**Published:** 2025-04-29

**Authors:** Chang Li, Kun Jiang, Shennan Pan, Chaogui Tang, Kai Wang

**Affiliations:** ^1^Department of Medical Laboratory, The Affiliated Huaian No.1 People’s Hospital of Nanjing Medical University, Huaian, Jiangsu, China; ^2^Department of Immunology and Rheumatology, The Affiliated Huaian No.1 People’s Hospital of Nanjing Medical University, Huaian, Jiangsu, China

**Keywords:** smoking, peptic ulcer disease, disability-adjusted life years, global health, trends, projections

## Abstract

**Objective:**

This study aims to analyze global trends in smoking-attributable peptic ulcer disease (PUD) disability-adjusted life years (DALYs) from 1990 to 2021 and project future trends to 2046.

**Methods:**

Data were obtained from the Global Burden of Disease Study 2021. We calculated age-standardized DALYs rates (ASDR) and estimated annual percentage changes (EAPC) for smoking-attributable PUD DALYs. Bayesian Age-Period-Cohort models were used to project future trends.

**Results:**

From 1990 to 2021, global smoking-attributable PUD DALYs decreased significantly, with the age-standardized rate declining from 35.4 to 9.4 per 100,000 (EAPC: −4.45%). High-income regions showed faster declines, while some low- and middle-income countries experienced slower progress or even increases. Gender disparities were observed, with males consistently showing higher ASDR. Projections suggest a continued global decline in smoking-attributable PUD DALYs to 2046, with persistent regional disparities. By 2046, the global ASDR is expected to decrease to approximately 3.2 per 100,000, with higher rates persisting in certain regions such as Kiribati (44.6 per 100,000) and Cambodia (45.1 per 100,000).

**Conclusion:**

While global smoking-attributable PUD DALYs have significantly decreased and are projected to continue declining, substantial regional and gender disparities persist. These findings underscore the need for targeted tobacco control interventions, particularly in high-risk regions and among vulnerable populations, to further reduce the global burden of smoking-attributable PUD.

## Highlights

- Global smoking-attributable PUD DALYs decreased significantly from 1990 to 2021.
- High-income regions showed faster declines in PUD DALYs than low/middle-income areas.
- Gender disparities persist, with males consistently showing higher ASDR than females.
- BAPC model projects continued global decline in smoking-attributable PUD DALYs to 2046.
- Regional disparities in PUD burden are expected to persist, necessitating targeted interventions.

## Introduction

Peptic ulcer disease (PUD) is a common gastrointestinal disorder primarily affecting the stomach and duodenum. Despite an overall declining trend in its incidence and mortality rates in recent decades, PUD remains a significant global public health concern (1). According to the Global Burden of Disease (GBD) 2019 study, approximately 32.9 million individuals worldwide were affected by PUD, resulting in 817,000 deaths (2). However, the disease burden of PUD is not uniformly distributed, with significant variations observed across different regions and populations (3).

Bleeding, perforation, or gastric outlet obstruction are the main complications of PUD (3). According to reports, perforated peptic ulcer is associated with short-term mortality in up to 30% of patients and morbidity in up to 50%. Although perforations are second to bleeding in frequency (about 1:6 ratio), they represent the most common indication for emergency surgery for PUD (4). The economic burden of PUD is substantial. Direct medical costs are estimated to range from $1,750 to $2,500 per patient annually in South Korea (5). The costs associated with complicated PUD are particularly high, with estimates ranging from $1,883 to $25,444 per patient (6). A US study conducted in 2000 revealed that PUD resulted in an average productivity loss of $606 per worker over a three-month period, highlighting the significant indirect costs through lost productivity (7). The impact on quality of life is equally significant. PUD patients consistently report markedly lower scores across multiple domains of health-related quality of life measures compared to the general population (8).

Smoking is a major risk factor for PUD, increasing both its incidence and mortality rates (9, 10). It influences PUD development and progression through various mechanisms (11, 12). Firstly, smoking stimulates gastric acid secretion, compromising the stomach’s mucosal defense barrier (13). Secondly, it reduces prostaglandin synthesis, which is crucial for maintaining gastric mucosal integrity (3, 14). Additionally, smoking decreases gastric mucosal blood flow, impairing the mucosa’s ability to repair itself (15). Notably, smoking may also indirectly increase the risk of PUD by affecting *Helicobacter pylori* infection rates and antibiotic resistance (16, 17). These mechanisms collectively not only increase the incidence of PUD but may also lead to more severe complications such as bleeding and perforation, thereby increasing mortality risk (18, 19).

Despite substantial progress in global tobacco control efforts and declining smoking rates in many countries, marked disparities persist across regions and populations (20). Smoking rates remain high in some low- and middle-income countries, potentially leading to uneven trends in smoking-attributable PUD disease burden worldwide (21).

To better understand and address the impact of smoking on PUD disease burden, a systematic analysis and projection of long-term trends is necessary. This approach allows for a comprehensive assessment of this public health issue’s evolution, providing scientific evidence for targeted prevention and intervention strategies. Moreover, predicting future trends is crucial for long-term public health planning and resource allocation (22).

This study utilizes the latest GBD 2021 data to analyze global trends in smoking-attributable PUD disability-adjusted life years (DALYs) from 1990 to 2021 and project future trends for the next 25 years. By focusing on differences across regions, income levels, and population characteristics, we aim to inform more effective tobacco control policies and PUD prevention strategies, ultimately reducing PUD-related disease burden, and improving global public health.

## Materials and methods

### Study design and data sources

Data on smoking-attributable PUD DALYs from 1990 to 2021 were extracted from the GBD 2021 database.^1^ The data were stratified by age, sex, region, and socio-demographic index (SDI). We analyzed overall trends as well as trends by gender, geographic region, and SDI groupings. For future trend predictions, we utilized GBD 2017–2100 global population forecasts to estimate population changes and project smoking-attributable PUD DALYs trends for the next 25 years.

### Statistical analysis

To assess trends in smoking-attributable PUD DALYs, we calculated age-standardized DALYs rate (ASDR) for 1990 and 2021. The estimated annual percentage change (EAPC) in ASDR was computed using a log-linear regression model, providing a summary measure of the average annual rate of change. We examined disparities in smoking-attributable PUD DALYs across regions and between sexes. Stratified analyses were conducted to identify regions and populations with the most significant changes in PUD DALYs.

For future trend predictions, we employed the Bayesian Age-Period-Cohort (BAPC) model to project the future burden of peptic ulcer disease. The model was implemented using age-specific mortality data from 1990 to 2021, with predictions extending to 2046. We structured our data into 18 age groups, ranging from 0 to 14 years to 95 + years, ensuring comprehensive coverage across the lifespan. The BAPC model incorporates several key components to ensure robust predictions. We applied second-order random walk priors for smoothing parameters, which helps maintain realistic transitions between adjacent time periods while accommodating potential non-linear trends in disease patterns. Age-standardized rates were calculated using WHO World Standard Population weights to facilitate international comparability. For model validation and uncertainty assessment, we conducted retrospective analyses using a 25-year prediction horizon. This approach allowed us to evaluate the model’s predictive accuracy by comparing projected values against observed data. We implemented rigorous convergence criteria and performed comprehensive diagnostic checks, including residual analysis and parameter stability assessments. Data visualization was performed using the “ggplot2” and “ggsci” packages. All statistical analyses were conducted using R (version 4.3.2). *p*-values less than 0.05 were considered statistically significant.

## Results

### Global trends in smoking-attributable peptic ulcer disease DALYs, 1990 vs. 2021

**Figure 1:**
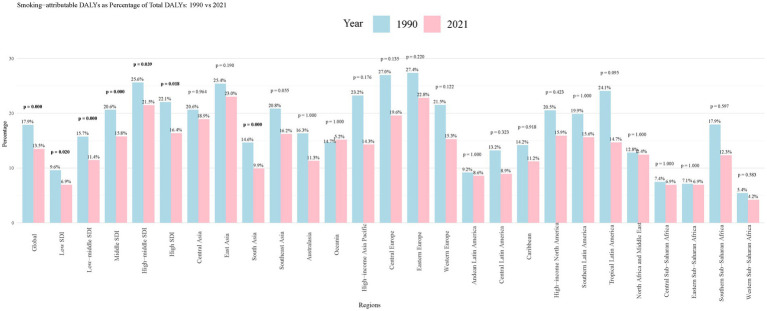
Smoking-attributable PUD DALYs as Percentage of Total DALYs: 1990 vs. 2021. PUD, peptic ulcer disease; DALYs, disability-adjusted life years.

Between 1990 and 2021, the proportion of smoking-attributable PUD DALYs to total DALYs decreased globally from 17.9 to 13.5% (*p* = 0.020) (Figure 1). This downward trend was observed across most regions, albeit to differing degrees. The high-income Asia Pacific region experienced the most significant decline (from 37.0 to 19.0%, *p* = 0.176), followed by East Asia (from 25.4 to 23.0%, *p* = 0.190). However, some regions such as Central Asia (from 22.1 to 21.5%, *p* = 0.018) and South Asia (from 20.0 to 18.9%, *p* = 0.964) showed slight increases or remained relatively stable. Notably, Oceania maintained a consistently low level with minimal change (from 11.3 to 15.2%, *p* = 1.000) over the 31-year period. These data reflect the overall effectiveness of global tobacco control measures while highlighting regional disparities in reducing smoking-related PUD burden.

### Trends in smoking-attributable peptic ulcer disease DALYs, 1990 to 2021

Table 1 illustrates significant changes in the global burden of smoking-attributable PUD from 1990 to 2021. The ASDR showed a substantial decrease from 35.4 (95% CI: 25.2–45.6) per 100,000 in 1990 to 9.4 (95% CI: 6.7–12.6) per 100,000 in 2021, with an EAPC of −4.45% (95% CI, −4.52% to −4.37%). Notable gender disparities were observed, with males consistently showing higher DALY rates compared to females, although both genders demonstrated declining trends over the study period.

**Table 1:** Smoking-attributable PUD DALYs and ASDR in 1990 and 2021, and its temporal trends from 1990 to 2021.

	1990		2021		1990–2021
Characteristics	DALYs	ASR	DALYs	ASR	EAPC
	No. ×10^3^ (95% UI)	No. (95% UI)	No. ×10^3^ (95% UI)	No. (95% UI)	No. (95% UI)
Overall	1500.8 (1070–1931.5)	35.4 (25.2–45.6)	817 (582.2–1093.1)	9.4 (6.7–12.6)	−4.45 (−4.52 to −4.37)
Sex					
Male	1344.9 (961.4–1733.8)	65.9 (47–85.5)	722.9 (520.9–970.9)	17.4 (12.5–23.4)	−4.46 (−4.53 to −4.39)
Female	156 (104.1–211.7)	7.3 (4.9–9.9)	94.1 (61.4–131)	2.1 (1.4–2.9)	−4.23 (−4.34 to −4.13)
Socio-demographic index					
Low	128.5 (84.6–173)	48.1 (31.6–65.4)	81 (51.9–115.8)	13.4 (8.6–19.1)	−4.37 (−4.52 to −4.23)
Low-middle	473 (319.5–644.6)	66.4 (44.7–91.3)	226.5 (151.2–318.3)	14.6 (9.8–20.5)	−5.09 (−5.22 to −4.96)
Middle	453.1 (327.3–596.9)	39.2 (28.1–51.8)	249 (174.4–342.6)	9 (6.3–12.4)	−4.72 (−4.81 to −4.64)
Middle-high	275.5 (199.4–350.4)	26.7 (19.3–33.9)	182 (131.1–236)	9.6 (6.9–12.4)	−3.62 (−3.75 to −3.49)
High	169.3 (121.2–218.2)	15.8 (11.4–20.4)	77.5 (54.1–101.8)	4.3 (3–5.7)	−4.36 (−4.59 to −4.13)
Region					
Central Asia	13.4 (9.7–16.7)	25.8 (18.7–32.1)	15.1 (10.7–20)	16.1 (11.4–21.3)	−2.45 (−2.81 to −2.09)
East Asia	443.3 (312.4–590.6)	47 (33.1–62.4)	211.5 (142.6–305.2)	9.9 (6.7–14.2)	−4.88 (−5 to −4.75)
South Asia	484.3 (326–672.2)	71 (47.6–100)	198.3 (123.5–288.4)	12.7 (7.8–18.4)	−5.75 (−5.92 to −5.59)
Southeast Asia	122.1 (79.6–174.2)	41.4 (27–59.6)	89.4 (62.1–124.9)	12.6 (8.8–17.6)	−4.03 (−4.18 to −3.89)
Australasia	3 (2.1–4)	12.8 (8.9–16.9)	0.7 (0.5–0.9)	1.4 (1–1.9)	−6.86 (−7.33 to −6.4)
Oceania	1.5 (1–2.2)	43 (28–60.6)	2 (1.3–2.9)	21.1 (13.6–31.3)	−2.44 (−2.51 to −2.36)
High-income Asia Pacific	30.8 (22.2–38.8)	15.2 (11–19.2)	10.4 (7.3–13.9)	2.8 (2–3.7)	−5.65 (−5.75 to −5.54)
Central Europe	48.5 (35.7–60.8)	32.8 (24.2–41)	32.3 (22.7–41.9)	17.3 (12.2–22.4)	−2.2 (−2.48 to −1.92)
Eastern Europe	75.3 (57.1–92.9)	27.5 (20.8–33.9)	82 (60.5–103.8)	26.4 (19.5–33.4)	−1.04 (−1.47 to −0.62)
Western Europe	83.6 (59.3–107.6)	15.4 (10.9–19.7)	24.1 (16.6–31.9)	3 (2.1–3.9)	−5.49 (−5.64 to −5.34)
Andean Latin America	4 (2.7–5.7)	18.3 (12.3–25.8)	2.2 (1.4–3.2)	3.6 (2.3–5.2)	−5.5 (−5.65 to −5.35)
Central Latin America	21 (15.1–26.8)	23.4 (16.8–30)	12.4 (8.3–16.7)	4.8 (3.3–6.6)	−5.61 (−5.87 to −5.34)
Caribbean	6.7 (4.7–9.1)	24.9 (17.3–33.7)	4.7 (3.1–6.5)	8.8 (5.8–12.2)	−3.66 (−3.91 to −3.41)
High-income North America	32.2 (22.4–42.8)	9.6 (6.7–12.7)	17.7 (12.1–23.9)	3.1 (2.1–4.1)	−3.91 (−4.31 to −3.51)
Southern Latin America	5.9 (4.3–7.5)	12.6 (9.1–16)	3 (2.1–3.9)	3.6 (2.5–4.7)	−3.91 (−4.43 to −3.39)
Tropical Latin America	26.2 (19–33.3)	25.6 (18.6–32.7)	18.7 (13–25.5)	7.1 (4.9–9.7)	−4.82 (−5.16 to −4.47)
North Africa and Middle East	40.7 (27.5–54)	21.5 (14.2–29)	26.6 (17.8–36.6)	5.3 (3.5–7.3)	−4.92 (−5.12 to −4.71)
Central Sub-Saharan Africa	5.2 (3.1–8.2)	19.4 (11.8–30.8)	7.4 (4.1–11.6)	10.4 (5.9–16.3)	−1.73 (−1.88 to −1.59)
Eastern Sub-Saharan Africa	31.2 (16.5–46.2)	33.2 (17.7–49)	33.9 (19.6–52.6)	14.6 (8.5–22.6)	−2.62 (−2.65 to −2.59)
Southern Sub-Saharan Africa	7.3 (5.2–9.7)	23.4 (16.6–31.2)	8.5 (5.8–11.6)	12.7 (8.6–17.2)	−1.64 (−2.01 to −1.28)
Western Sub-Saharan Africa	14.6 (9.5–20.4)	14 (9.2–19.6)	16.1 (10.5–22.4)	6.3 (4.1–8.7)	−2.66 (−2.85 to −2.46)

ASRs were calculated using the WHO 2000–2025 World Standard Population. The formula is ASR = Σ(w[i] * r[i])/Σw[i], where w[i] is the standard population weight for age group i, and r[i] is the age-specific rate. This standardization allows for fair comparisons across populations with different age structures and over time.

EAPC was calculated to quantify temporal trends in ASRs from 1990 to 2021. We used the linear regression model ln(ASR) = β0 + β * (year – 1990) + ε, where EAPC = (e^β – 1) * 100%. The 95% uncertainty intervals (UIs) were calculated as [(e^(β – 1.96 * SE) – 1) * 100%, (e^(β + 1.96 * SE) – 1) * 100%]. A positive EAPC indicates an increasing trend, while a negative value indicates a decreasing trend. The trend is considered statistically significant if the 95% UI does not include zero.

PUD, peptic ulcer disease; ASR, age standardized rate; UI, uncertainty interval; EAPC, estimated annual percentage change; UI, uncertainty interval.

Analysis of EAPC values revealed substantial regional heterogeneity in PUD DALYs trends. High-income regions demonstrated favorable trends (EAPC = −2.1, 95% CI: −2.4, −1.8), while middle-income regions showed varying patterns. Eastern Europe exhibited rapid improvement (EAPC = −2.8, 95% CI: −3.1, −2.5), whereas Southeast Asia showed slower progress (EAPC = −0.9, 95% CI: −1.2, −0.6). Low-income regions, particularly Sub-Saharan Africa, demonstrated the least favorable trends (EAPC = −0.3, 95% CI: −0.5, −0.1).

Across SDI strata, low-middle SDI regions showed the most rapid decline (EAPC: −5.09%). Regional analysis revealed that Australasia (−6.86%) and South Asia (−5.75%) exhibited the most significant improvements, while Oceania (−2.44%) and Eastern Europe (−1.04%) showed slower rates of decline. Central Asia, Eastern Europe, and sub-Saharan Africa maintained relatively slower rates of improvement compared to other regions.

### Regional and country-specific trends, 1990 to 2021

**Figure 2:**
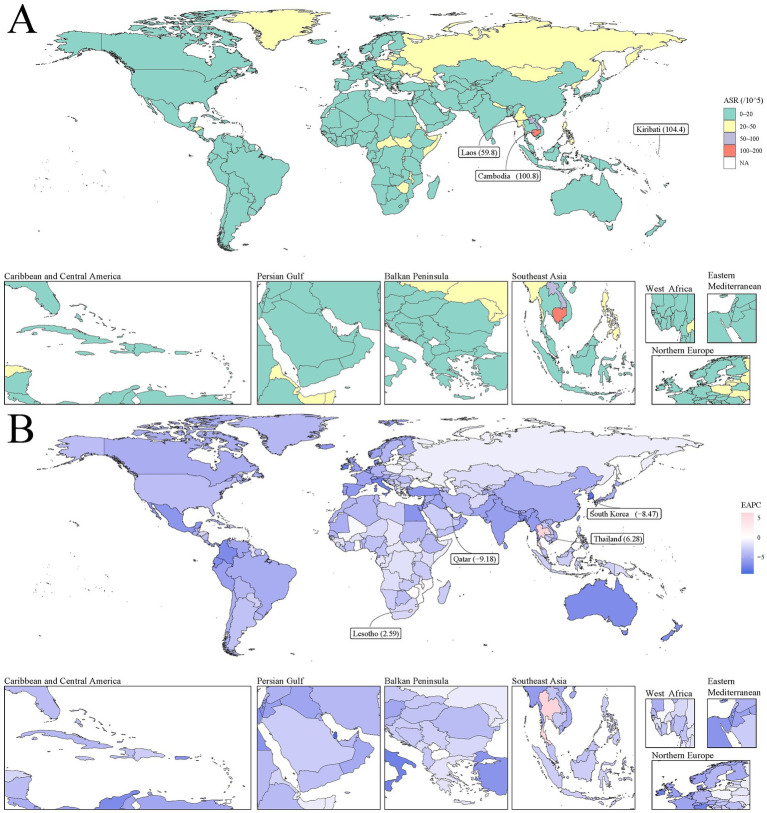
The global disease burden of smoking-attributable PUD DALYs for both sexes in 204 countries and territories. **(A)** The ASDR of PUD in 2021; **(B)** The EAPC of smoking-attributable PUD DALYs from 1990 to 2021. Countries with an extreme number of cases/evolution were annotated. PUD, peptic ulcer disease; DALYs, disability-adjusted life years; ASR, age-standardized rate; EAPC, estimated annual percentage change.

Most countries exhibited relatively low DALY rates (0–20 per 100,000). However, some countries and regions showed notably higher burdens, particularly in Southeast Asia (Figure 2A). Cambodia (100.8 per 100,000), Laos (59.8 per 100,000), and Kiribati (104.4 per 100,000) had exceptionally high DALY rates. The majority of countries demonstrated negative EAPC values, indicating a general decline in PUD burden over the 31-year period (Figure 2B). South Korea (−8.47%) and Qatar (−9.18%) showed the fastest rates of decline. However, a few countries such as Thailand (0.28%) and Lesotho (2.59%) experienced positive EAPC values, suggesting an increase in PUD burden in these regions.

### Relationship between ASMR and socio-demographic index (SDI)

**Figure 3:**
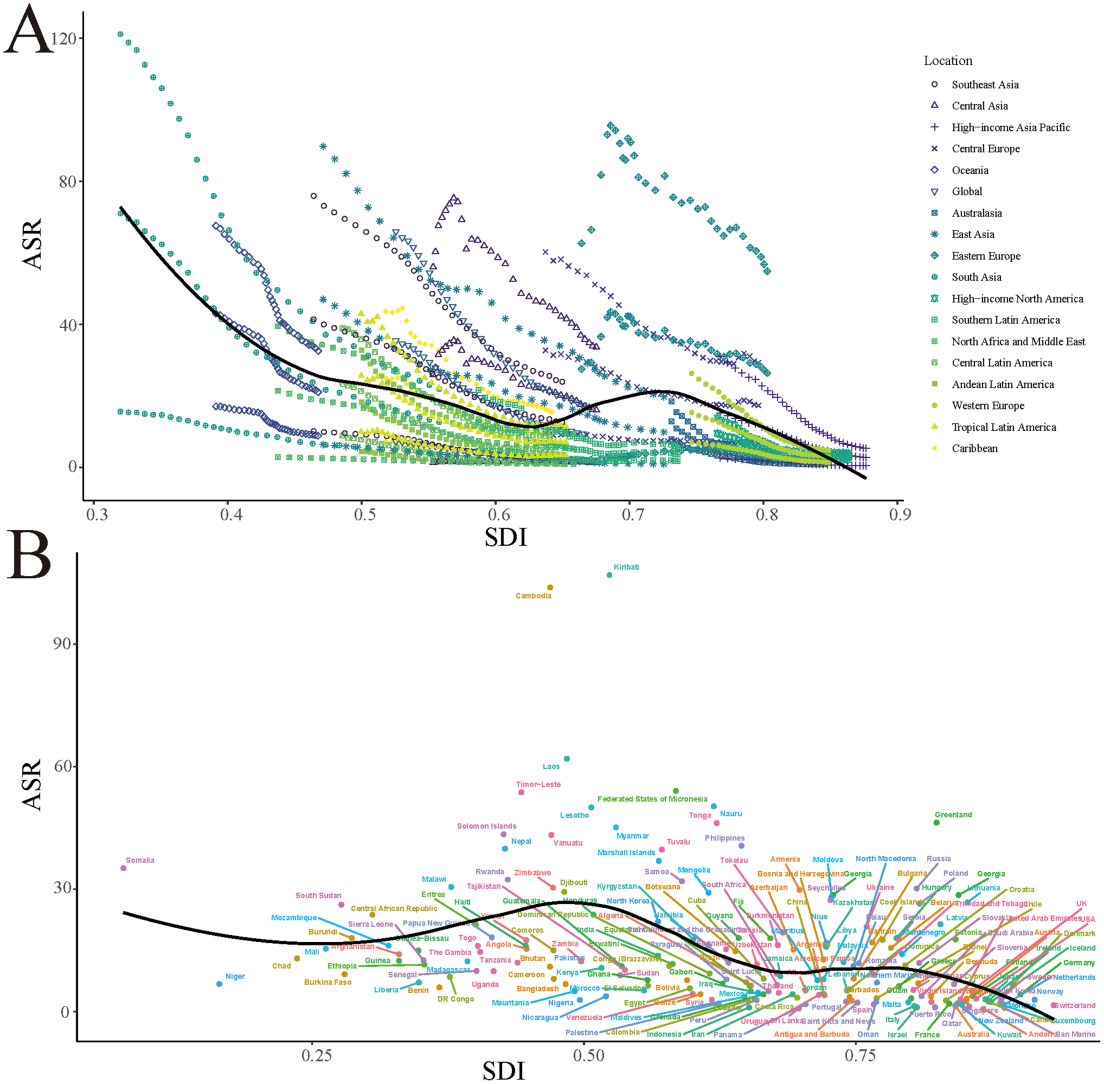
Smoking-attributable PUD ASDR in different GBD regions **(A)** and 204 countries and territories **(B)** by SDI, 1990–2021. Expected values based on Socio-demographic index and disease rates in all locations are shown as the black line. PUD, peptic ulcer disease; DALYs, disability-adjusted life years; ASR, age-standardized rate; GBD, Global Burden of Disease; SDI, socio-demographic index.

Overall, DALY rates showed a negative correlation with SDI, indicating that improvements in socioeconomic development are associated with a reduction in PUD disease burden (Figure 3A). However, this relationship exhibited marked heterogeneity across regions. East Asia and South Asia demonstrated higher DALY rates at lower SDI values, while high-income Asia Pacific regions maintained relatively low DALY rates even at higher SDI values. Notably, Central Asia and Eastern Europe maintained higher DALY rates at medium SDI levels, reflecting the importance of region-specific factors in shaping disease burden.

While most countries showed a downward trend in DALY rates with increasing SDI, some countries such as Kiribati, Cambodia, and Laos maintained exceptionally high DALY rates at relatively low SDI levels. This deviation from the expected trend (indicated by the black line in the figure) emphasizes the importance of factors beyond SDI, such as healthcare policy implementation, cultural practices, and access to medical resources, in shaping PUD disease burden (Figure 3B).

### Trends in smoking-attributable peptic ulcer disease DALYs, 2022 to 2046

**Figure 4:**
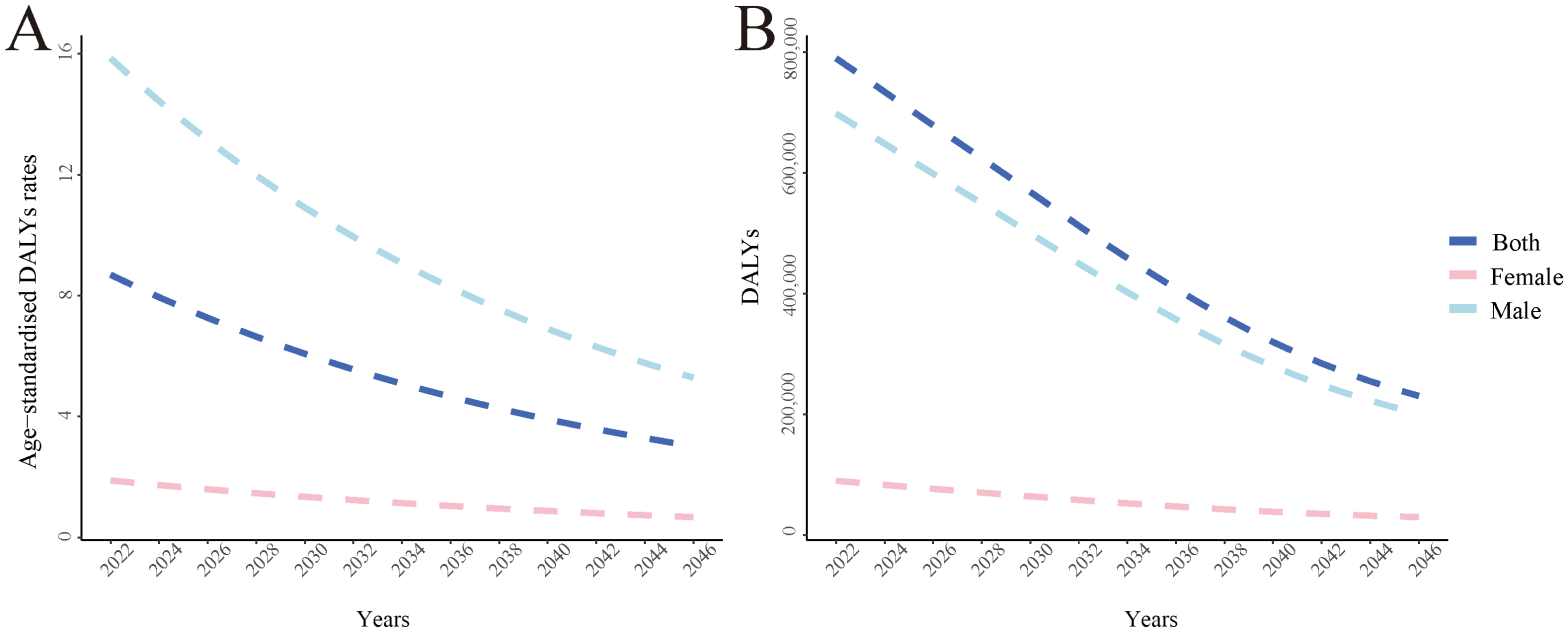
The global disease burden of smoking-attributable PUD DALYs ASDR predicted by BAPC model in 2046. **(A)** Smoking-attributable PUD age-standardized of DALYs rate predicted by BAPC model in 2046; **(B)** Smoking-attributable PUD DALYs predicted by BAPC model in 2046. PUD, peptic ulcer disease; DALYs, disability-adjusted life years; BAPC, Bayesian Age-Period-Cohort.

BAPC model projections indicate a continuing downward trend in both ASDRs and total DALYs (Figure 4). This trend is observed across males, females, and the total population, although the magnitude of decline varies by gender. Specifically, the ASDR for males is projected to decrease from approximately 15.5 per 100,000 in 2022 to about 5.5 per 100,000 in 2046, while for females, it is expected to decline from about 1.8 per 100,000 to 0.8 per 100,000. The overall ASDR is projected to decrease from about 8.5 per 100,000 to 3.2 per 100,000. Regarding the total number of DALYs, those attributable to smoking-related PUD for males are expected to decrease from about 700,000 in 2022 to 230,000 in 2046, while for females, the number is projected to decline from about 80,000 to 30,000. The total number of DALYs is expected to decrease from about 780,000 to 260,000. Notably, while both sexes show declining trends, male DALY rates and total DALYs consistently remain significantly higher than those of females, with this gender disparity persisting throughout the projection period. These findings highlight the importance of gender-specific intervention strategies in reducing smoking-related PUD burden.

**Figure 5:**
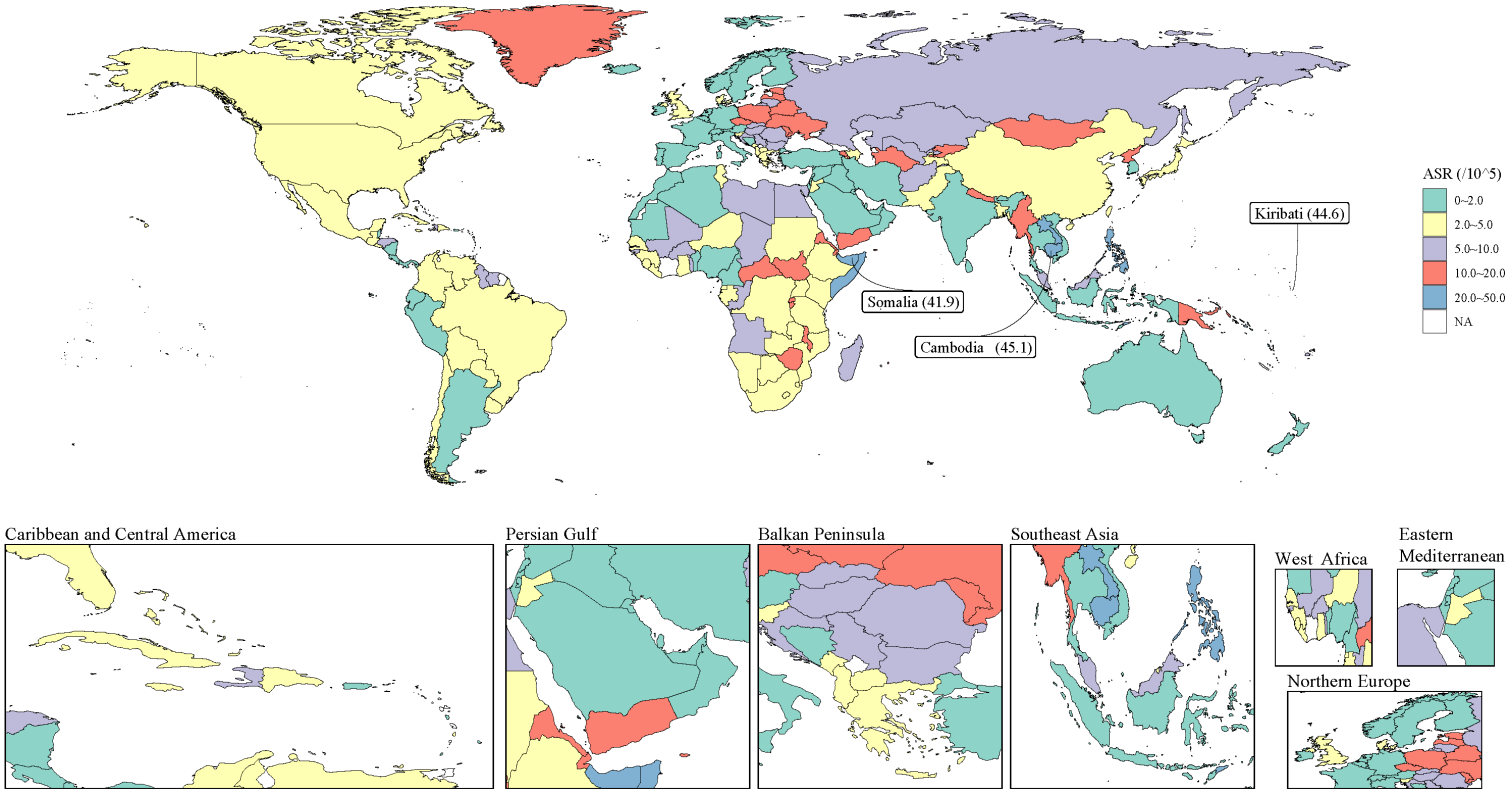
Smoking-attributable PUD DALYs predicted by BAPC model in 2046. Countries with an extreme number of cases/evolution were annotated. PUD, peptic ulcer disease; DALYs, disability-adjusted life years; BAPC, Bayesian Age-Period-Cohort.

Most countries and regions are projected to maintain relatively low ASDRs (0–5.0 per 100,000), indicating significant improvements in PUD burden globally. However, the projections also reveal persistent regional disparities. Notably, some countries are expected to maintain relatively high disease burdens. Among these, Kiribati (44.6 per 100,000), Cambodia (45.1 per 100,000), and Somalia (41.9 per 100,000) are projected to have ASRs significantly higher than the global average and are specifically highlighted in the figure. This finding emphasizes the need for additional intervention measures and resource allocation in these high-risk areas in the future. Furthermore, Eastern Europe, Central Asia, and parts of Africa are expected to maintain medium levels of ASR (5.0–10.0 per 100,000), indicating ongoing challenges in reducing PUD burden in these regions. In contrast, North America, Western Europe, Australia, and parts of South America are projected to achieve the lowest ASR levels (0–2.0 per 100,000), reflecting the potential long-term effectiveness of tobacco control and PUD prevention efforts in these areas (Figure 5).

## Discussion

Our findings demonstrate a significant global decline in smoking-related PUD burden, with complex patterns varying across regions and socioeconomic strata. The observed decline patterns reflect the combined effects of improved healthcare delivery and tobacco control measures. However, this progress is not evenly distributed. High-income regions demonstrate more rapid improvements, particularly in ASDR reduction, suggesting that comprehensive healthcare systems and strict tobacco control policies can effectively reduce disease burden. In contrast, some low- and middle-income countries showed slower progress or even upward trends, highlighting the persistence of global health inequalities.

Our study also revealed a complex relationship between PUD DALYs and the SDI. While the overall trend shows a decrease in PUD DALYs with increasing SDI, this relationship is not linear. Some medium-SDI countries exhibited higher than expected PUD burdens, possibly reflecting unique challenges faced by these countries during rapid economic development, such as lifestyle changes due to urbanization and increased environmental pollution (23).

Several underlying mechanisms contribute to these trends. The decreasing smoking prevalence, particularly in developed regions, has directly impacted PUD incidence. The gender disparity in DALY rates, while persistent, shows signs of narrowing, reflecting changing social norms and smoking patterns. The varying rates of decline across SDI strata (fastest in low-middle SDI regions at −5.09%) suggest that economic development alone does not determine progress in reducing disease burden. Policy effectiveness appears to vary significantly across regions. While Australasia (−6.86%) and South Asia (−5.75%) demonstrate successful implementation of tobacco control measures, slower progress in regions like Eastern Europe (−1.04%) and Oceania (−2.44%) suggests the need for policy refinement. The effectiveness of existing interventions appears to be modulated by local healthcare capacity, cultural factors, and socioeconomic conditions.

Gender disparity is another noteworthy aspect. Our findings show PUD DALYs are consistently higher in males than in females, which can be attributed to multiple factors. Biological mechanisms play a crucial role - males generally have higher basal acid secretion and different patterns of inflammatory responses compared to females. Studies have shown that testosterone may enhance gastric acid secretion, while estrogen appears to have protective effects on gastric mucosa through various molecular pathways (24). Smoking behaviors significantly contribute to these gender differences. Males typically exhibit higher smoking rates and more intensive smoking patterns globally, although this gap has been narrowing in recent decades (21). Male smokers are more likely to be heavy smokers and start smoking at an earlier age, potentially leading to more severe gastric complications (25). Healthcare utilization patterns also differ between genders. Research indicates that women are more likely to seek medical attention for early symptoms and adhere to preventive care recommendations (26). This behavioral difference may result in earlier detection and treatment of PUD in females, potentially leading to better outcomes. These findings have important implications for intervention strategies. Gender-specific approaches to smoking cessation programs and PUD prevention may be more effective than general population strategies.

Our prediction model indicates that global PUD DALYs will continue to decline over the next 25 years, but regional disparities may persist. This prediction provides important evidence for formulating long-term public health strategies. However, it should be noted that this prediction may not fully account for the potential impact of emerging factors such as the proliferation of new tobacco products and the influence of global climate change on dietary patterns (27–29). The emergence of novel tobacco products, particularly electronic cigarettes and heated tobacco products, introduces new uncertainties into future PUD burden projections. While our model captures traditional smoking patterns, the rapid adoption of these alternative nicotine delivery systems may reshape future risk landscapes. Recent experimental evidence in mice demonstrated that compared to conventional cigarette smoke, e-cigarette aerosol exposure resulted in significantly fewer adverse effects on gastrointestinal indicators, with notably less damage to oral mucosa after 10 weeks of exposure (30).

Based on these findings, we propose the following policy recommendations: (1) Develop differentiated tobacco control strategies tailored to countries with different SDI levels. For instance, high-SDI countries may focus more on regulating new tobacco products, while low-SDI countries may need more basic education and price intervention measures (31). (2) Strengthen cross-sector collaboration: The reduction of PUD DALYs depends not only on tobacco control but is also closely related to overall healthcare levels and dietary habits. Therefore, coordinated efforts from health, education, agriculture, and other sectors are necessary (32). (3) Focus on vulnerable populations: Our study shows that certain groups (such as males in low-SDI countries) face higher PUD risks. Developing targeted intervention measures for these high-risk groups is crucial (33). (4) Enhance global cooperation: Given the significant disparities in the global distribution of PUD DALYs, strengthening international cooperation and sharing successful experiences and best practices become particularly important (34). (5) Invest in long-term monitoring and research: Continuous monitoring of PUD DALY trends and in-depth study of influencing factors are essential for timely policy adjustments (35, 36).

The strengths of this study lie in its use of the latest GBD data and advanced BAPC models for trend prediction. However, the study also has limitations. First, the completeness and accuracy of PUD data vary substantially across regions. High-income countries generally maintain robust vital registration systems and healthcare databases, while in low- and middle-income countries (LMICs), data quality challenges are more pronounced. These variations include incomplete vital registration coverage, limited healthcare facility reporting, and diagnostic uncertainties. In regions with limited healthcare access, PUD cases might be systematically underreported, particularly for mild to moderate cases. Many low- and middle-income countries lack standardized coding systems for gastrointestinal diseases, potentially leading to misclassification. Second, our prediction model may not fully capture all factors that could influence future trends. Global events such as pandemics can significantly alter smoking behaviors and healthcare resource allocation, which in turn affect PUD burden (37). Furthermore, temporal trends in some regions might reflect improvements in surveillance systems rather than true changes in disease burden, making it challenging to interpret apparent changes in disease patterns. These data quality issues and model limitations particularly impact our projection estimates. While our analyses present future PUD DALYs trends, these estimates assume current data quality patterns persist and may not fully account for unprecedented global events or rapid changes in healthcare systems. Therefore, our findings should be interpreted with appropriate caution, particularly for regions with known data quality issues.

In conclusion, this study provides new perspectives for understanding the dynamic changes in global PUD disease burden, emphasizing the importance of developing targeted and forward-looking public health strategies to address this ongoing global health challenge.

## Data Availability

The original contributions presented in the study are included in the article/supplementary material, further inquiries can be directed to the corresponding authors.

## Footnotes

^1^ https://ghdx.healthdata.org/gbd-2021
